# Evidence for cephalic magnetic map receptors in sea turtles

**DOI:** 10.1242/jeb.252113

**Published:** 2026-05-28

**Authors:** Dana S. Lim, Kayla M. Goforth, Catherine M. F. Lohmann, Kenneth J. Lohmann

**Affiliations:** Department of Biology, University of North Carolina at Chapel Hill, Chapel Hill, NC 27599, USA

**Keywords:** Magnetoreception, Orientation, Navigation, Sensory receptors

## Abstract

Although the ability to sense Earth's magnetic field is phylogenetically widespread, receptors for the magnetic sense have not been identified unequivocally in any animal. Because magnetic fields penetrate biological tissue, magnetoreceptors could hypothetically exist anywhere in the body. To investigate the location of magnetoreceptors in juvenile loggerhead sea turtles (*Caretta caretta*), we attached a weak magnet, which generated a magnetic disturbance over a small localized area, to three anatomical regions (head, mid-body and posterior). We then observed responses of turtles to magnetic map cues they had been conditioned to associate with food. Responses to the food-associated field decreased significantly when a magnet was attached to the head, but no such disruption occurred when the magnet was placed at the other locations. The results provide the first evidence for cephalic magnetoreceptors in turtles and provide a simple methodology that can be used to help localize receptors in other magnetoreceptive animals.

## Teaser

**Summary:** Behavioral experiments provide evidence that receptors for the magnetic map sense in loggerhead sea turtles are located in the head.

## INTRODUCTION

Diverse animals, including invertebrates, fishes, amphibians, mammals, sea turtles, and birds, possess the ability to sense Earth's magnetic field and use it in orientation and navigation (reviewed by Goforth and Merlin, 2025; Naisbett-Jones and Lohmann, 2022; Phillips and Diego-Rasilla, 2022; Begall et al., 2013; Lohmann et al., 2012; Wiltschko and Wiltschko, 1995). In some animals, magnetic information can be used both as a ‘compass’ to obtain directional information and a ‘map’ to obtain positional information (Wiltschko and Wiltschko, 2005; Lohmann et al., 2022). Despite the prevalence of magnetic sensitivity in animals, magnetoreceptors have not been localized or identified with certainty in any animal and the mechanisms underlying magnetoreception remain enigmatic (Nordmann et al., 2017).

The search for magnetoreceptors is made especially difficult because magnetic fields pass unimpeded through biological tissue, meaning that receptors might, in principle, be located anywhere in the body (Johnsen and Lohmann, 2005, 2008). Indeed, numerous locations have been proposed, including the eyes (Ritz et al., 2000; Caspar et al., 2020), semicircular canals (Nimpf et al., 2019; Nordmann et al., 2026), pineal gland (Deutschlander et al., 1999) and nose (Walker et al., 1997). Non-cephalic locations have also been suggested, including the lateral line of fishes (Moore et al., 1990), the abdomen of bees (Walker and Bitterman, 1989), the ventral cephalothorax of lobsters (Lohmann, 1984) and dispersed locations in the peripheral tissues of a mollusc (Pavlova et al., 2011). Given the difficulty of locating magnetoreceptors, techniques that can help narrow the search are needed.

Here, we investigate the location of magnetoreceptors in juvenile loggerhead sea turtles (*Caretta caretta*). Sea turtles are iconic migratory animals that rely at least partly on magnetic compass and map senses to guide lengthy oceanic journeys (Lohmann et al., 1997, 2012). The magnetic map sense serves to direct young turtles along their trans-oceanic migratory pathways and helps older turtles identify ecologically important areas such as feeding sites by allowing animals to perceive ‘magnetic signatures’ that mark different oceanic regions (Lohmann et al., 2001, 2004, 2022). Two geomagnetic parameters that vary geographically appear to comprise these magnetic signatures: the inclination angle (angle at which magnetic field lines intersect Earth's surface) and the intensity (strength) of the magnetic field (Lohmann and Lohmann, 1994, 1996; Lohmann et al., 2001; Putman et al., 2011; Goforth et al., 2025).

A recently developed behavioral assay allows the magnetic map sense to be decoupled from the magnetic compass (Goforth et al., 2025; Mackiewicz et al., 2025). In this technique, turtles are conditioned to associate a magnetic signature with a food reward. Turtles demonstrate recognition of the rewarded magnetic signature by exhibiting greater amounts of a food-anticipatory behavior (known as the ‘turtle dance’) in their rewarded field, compared with a field in which they were not rewarded (Goforth et al., 2025). Because the assay does not involve a directional response and requires only the detection of magnetic map cues, it provides a way to study the magnetic map sense without involving the magnetic compass.

To investigate the location of magnetic map receptors in turtles, we first conditioned turtles using this technique and then tested turtles in their rewarded field with small weak magnets placed on different parts of the body. We reasoned that if magnetoreceptors exist in those specific anatomical areas, then magnetoreception would be disrupted. These targeted magnetic distortions were used to assess where the receptors for the magnetic map sense reside.

## MATERIALS AND METHODS

### Animals

Loggerhead sea turtles [*Caretta caretta* (Linnaeus 1758)] were collected from nests on Bald Head Island, North Carolina, USA in August–September 2019. A total of 16 hatchlings were collected in pairs from eight different nests. The turtles were transported to the University of North Carolina (UNC) in Chapel Hill, North Carolina, USA, and were maintained there for the duration of the experiments. Turtles were housed in individual tanks in a re-circulating artificial seawater system with water temperature maintained between 26°C and 28°C. Turtles were fed gelatin diet (Mazuri^®^) and squid. Experiments were conducted in July 2020, when the turtles were between 11 and 12 months old and weighed approximately 985–1430 grams. Straight carapace length was 18.4–21.0 cm, straight carapace width was 14.5–17.3 cm, head length from nose tip to base of skull was 4.7–6.0 cm, and head width was 3.5–4.0 cm. All collection and research activities were approved by the North Carolina Wildlife Resources Commission (permit ST44) and the UNC Institutional Animal Care and Use Committee (protocol 20-248.0).

### Conditioning set-up and procedure

The conditioning set-up and assay have been described previously (Goforth et al., 2025). Turtles were conditioned in a magnetic coil system consisting of three independent four-coil systems arranged orthogonally. Each four-coil system (Alldred and Scollar, 1967) was controlled by a separate power supply (BK Precision Model 1550 DC).

The magnetic coil system was used to replicate magnetic fields that existed in two target areas: one near Virginia, USA and a second near Newfoundland, Canada (Table S1). Magnetic fields for these locations were derived from estimates provided by the International Geomagnetic Reference Field (IGRF) model 13 in spring 2020. Magnetic parameters were measured with a Meda tri-axial magnetometer (model FVM-400) in the central area within the coil where turtles were positioned during experiments (Table S1).

For both the conditioning and testing phases of the study, turtles were placed individually into 60 liter cylindrical buckets (diameter 55 cm) filled with artificial sea water to a depth of 30 cm. Buckets were arranged in the center of a plywood platform inside the magnetic coil system, where the field generated by the coil system was most uniform.

Conditioning took place over 2 months, between March and May 2020. Turtles were repeatedly exposed to the two different magnetic signatures, one in which they received food (rewarded field) and one in which they did not (unrewarded field). In these experiments, half of the turtles (*n*=8) were assigned a rewarded magnetic field that replicated one existing near Virginia, USA and an unrewarded field replicating one that exists near the coast of Newfoundland, Canada (Table S1). The other half of the turtles (*n*=7) experienced the Newfoundland field as their rewarded field and the Virginia field as their unrewarded field. One additional turtle was rewarded and tested in the Newfoundland field but excluded from final analyses because of the onset of a health condition that reduced food motivation. Turtles alternated daily between experiencing the field in which they received food and the field in which they did not. Four turtles from each group were conditioned at a time, and each conditioning session began with a 20 min acclimation period in a magnetic field similar to the one that exists in the facility where the turtles were housed (Table S1). After the acclimation period, the group was exposed to one of the two magnetic field signatures for 40 min. During sessions in which turtles received food, turtles were fed 5–15 min after the field was changed to their rewarded field. During sessions in which turtles were not fed, turtles remained in their unrewarded field for 40 min and did not receive food at any point that day.

### Behavioral trials

After the 2 month conditioning period, each turtle was tested in both their rewarded and unrewarded magnetic fields in randomized order. These initial tests allowed us to determine whether turtles had learned to discriminate between these fields, based on the amount of turtle dancing displayed in each. For these trials, each turtle was placed in its bucket and tested alone inside the magnetic coil system, with no other turtles present in the room. As with conditioning sessions, turtles began each test period with 20 min in the acclimation field. The magnetic field was then changed to the rewarded or unrewarded field for another 20 min. Each test period was recorded using a camera (GoPro^®^) affixed to the magnetic coil system above the bucket. No observers were present in the room during experiments. Turtles were tested every other day, on days when they would normally have been fed in accordance with the established conditioning pattern. Each turtle was tested at the same time of day in all treatments and the temperature of the water was measured before and after each trial, to ensure that both factors remained consistent. At the end of all trials each day, turtles that had been tested that day were fed in their rewarded magnetic field to reinforce the conditioned response. This reinforcement continued until the magnet treatment experiment was completed.

Two months after initial conditioning, turtles underwent testing in their rewarded magnetic field under four different magnet placement treatments described in the section below. These trials took place within the 4 month period during which turtles are known to retain their rewarded responses (Goforth et al., 2025), even without the further conditioning reinforcement that turtles in our study received. Testing in the rewarded field with magnet treatments followed the same protocol as the initial tests of learning described above.

### Magnet treatments

Each turtle underwent behavioral trials in their rewarded field under four treatments, with the sequence randomized for each turtle. The treatments were: (1) no magnet attached (control); (2) magnet attached to the head (approximately between the eyes); (3) magnet attached to mid-carapace (vertebral scute 3); and (4) magnet attached to posterior carapace (vertebral scute 5).

**Fig. 1.**
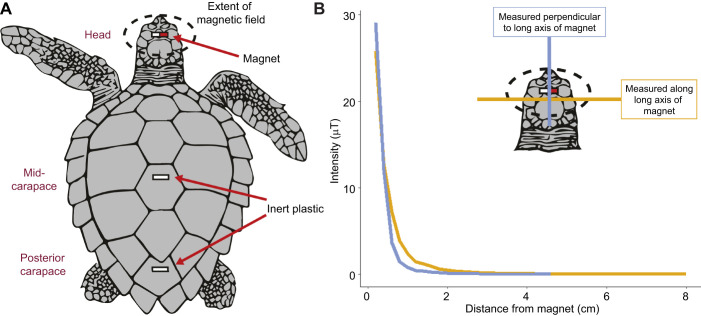
**Magnet placement on juvenile loggerhead sea turtle (*Caretta caretta*) and measurements of magnet strength.** (A) Diagram showing a turtle with a magnet attached to its head (north pole, indicated in red, pointed right) and magnetically inert plastic pieces of equal weight attached at the mid-carapace and posterior carapace positions. In the mid-carapace and posterior carapace treatments, the magnet was affixed to the target location and plastic pieces attached at the other two sites. In the no-magnet control, plastic pieces were placed at all three positions. The dotted line around the head illustrates the approximate extent of the magnetic field produced by the magnet. Velcro pieces used for attachment are not shown. (B) Strength of magnetic field as a function of distance from the magnet. Orange indicates the intensity of the field measured along a line defined by the long axis of the magnet. Blue indicates the intensity measured along a line perpendicular to the long axis of the magnet. The alignment of the gaussmeter probe is shown on the graphic of the turtle head. The gaussmeter was zeroed prior to measurements; thus, intensity values represent the difference from the ambient magnetic field.

For every trial, each turtle had a small piece of Velcro^®^ attached with cyanoacrylate adhesive to three positions on its body: top of the head, middle of the carapace, and posterior carapace. An object was attached with Velcro at every position (Fig. 1A). In treatment 1 (control), small, non-magnetic plastic pieces were attached at all three positions. In treatments 2–4, a magnetic stir bar (Chemglass, 2×5 mm) was attached to the treatment's target area and plastic pieces of equivalent weight, size and color were attached to the other two locations. For example, in treatment 2, which tested disruption to the head, the magnetic stir bar was attached at the head position and plastic pieces were affixed to the mid-carapace and posterior carapace positions (Fig. 1A). The magnet was always attached perpendicular to the body length with the north pole of the magnet on the right side of the body. The non-magnetic plastic pieces were attached to the positions without the magnet to ensure that having additional weight at any of the three positions did not affect behavior. The Velcro, magnet and plastic pieces were attached 20 min before the start of each trial. At the end of each trial, the cyanoacrylate adhesive and Velcro were removed easily, as the adhesive became brittle after being submerged in saltwater.

**Fig. 2.**
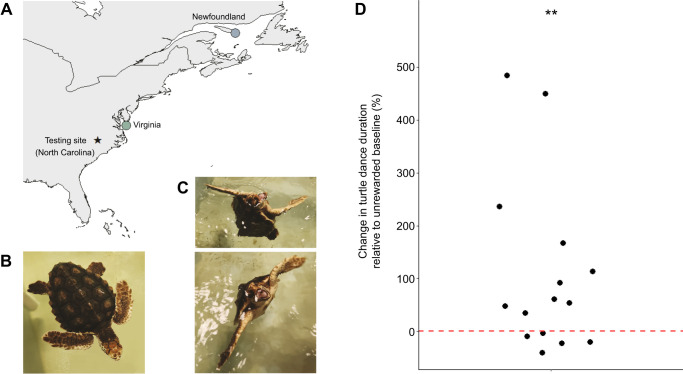
**Locations used for map assay, examples of turtle dance behavior and initial test of magnetic field learning.** (A) Map showing the testing site in North Carolina, USA (black star) and the two approximate locations, Virginia, USA (green circle) and Newfoundland, Canada (blue circle) whose magnetic signatures were replicated in the map conditioning assay. (B) Juvenile loggerhead turtle at rest, not displaying turtle dance behavior. (C) Juvenile loggerhead turtle displaying turtle dance behavior, with the body tilted vertically, head lifted out of water and mouth open, accompanied by front flipper paddling. Photos courtesy of Peter A. Lim. (D) Percentage change in turtle dance behavior in the rewarded field relative to the unrewarded field. In the rewarded field, turtles displayed a percentage change significantly greater than zero (one-tailed Wilcoxon signed-rank test, *w*=104, *P*=0.005, *n*=15). Dashed red line represents 0% change. Black dots represent changes in an individual's response. **P<0.01.

The strength and spatial extent of the magnetic field generated by the magnetic stir bar were measured with a gaussmeter (Magnetic Instrumentation model 912). The gaussmeter probe was moved stepwise along a line defined by the long axis of the magnet for one set of measurements and perpendicular to the line defined by the long axis of the magnet for the other. At 0.2 cm distance from the magnet, the highest intensity measured was 29.1 μT, which then exponentially decreased until becoming negligible at approximately 3 cm from the magnet (Fig. 1B). Thus, the magnetic field disruption due to the magnet was limited to the anatomical area being tested. At the same time, this weak magnet should still have been of sufficient strength to impair the turtle's ability to distinguish between the two selected Earth-strength fields (Fig. 2A; Table S1), if positioned near the receptors.

### Data analysis

For each 20 min experimental period, we recorded the amount of time a turtle spent exhibiting turtle dancing (Goforth et al., 2025). Turtle dancing is a food-anticipatory behavior characterized by some or all of the following: tilting the body vertically, raising the head out of water, opening the mouth, alternating movement of the front flippers and occasionally spinning in place (Fig. 2B,C). Two or more trained observers, blind to the treatment, scored each video using BORIS (Behavioral Observation Research Interactive Software). The time each turtle spent dancing in each treatment was averaged between the individual observations (Table S4).

Initial conditioning results from these 16 turtles were originally reported in an earlier study (Goforth et al., 2025) but, as noted previously, one of these turtles had to be excluded from subsequent magnet experiments because of health issues. To verify that the subset of turtles used in the magnet study had learned to discriminate between their rewarded and unrewarded fields, we calculated percentage change in turtle dance behavior in the rewarded field relative to the unrewarded field for each individual, with the same equation used in Goforth et al. (2025):
(1)

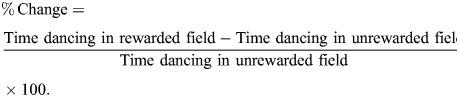


Because we expected turtles to exhibit more turtle dancing in the rewarded field compared with the unrewarded field, we used a one-tailed Wilcoxon signed-rank test to assess whether the percentage change in turtle dancing was greater than zero, as predicted.

To determine if each magnet treatment affected magnetic field detection compared with the no-magnet control, we calculated percentage change in turtle dance behavior in each of the three magnet treatments relative to the no-magnet control treatment for each individual:
(2)

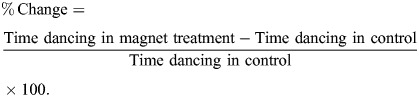


This analysis allowed us to assess the magnitude of change each turtle demonstrated in each treatment compared with its own baseline behavior in the control. In this case, if the magnet in a given treatment had an effect, we expected the amount of turtle dancing to decrease significantly relative to the no-magnet control. This was assessed using a one-tailed Wilcoxon signed-rank test.

We also assessed the effect of magnet placement on duration of turtle dancing using a linear mixed-effects model [R packages ‘lme4’ (https://CRAN.R-project.org/package=lme4; Bates et al., 2015), ‘lmerTest’ (https://CRAN.R-project.org/package=lmerTest; Kuznetsova et al., 2017)], with magnet placement as a fixed effect and no-magnet control as the intercept. Turtle ID was included as a random effect (Table S2). We confirmed that the data met model assumptions using the R package ‘performance’ (https://CRAN.R-project.org/package=performance). We used *post hoc* pairwise comparisons to determine whether there were significant pairwise differences between treatments using ‘emmeans’ (https://CRAN.R-project.org/package=emmeans). Given our small sample size (*n*=15), we performed *post hoc* power analyses to contextualize the sensitivity of our study using R packages ‘rstatix’ (https://CRAN.R-project.org/package=rstatix), ‘coin’ (https://cran.r-project.org/package=coin; Hothorn et al., 2008) and ‘pwr’ (https://CRAN.R-project.org/package=pwr). All statistical tests and visualization were completed with RStudio version 4.4.0 (r-project.org).

## RESULTS AND DISCUSSION

After conditioning, turtles were presented with their rewarded or unrewarded field to determine whether they could distinguish between them (Fig. 2A). In the rewarded field, turtles displayed a significant percentage increase in time spent turtle dancing relative to the unrewarded field (Fig. 2B–D; one-tailed Wilcoxon signed-rank test, *w*=104, *P*=0.005). These results imply that turtles learned to associate food with a particular magnetic signature and could distinguish that field from an unrewarded field, providing a foundation for subsequent experiments.

**Fig. 3.**
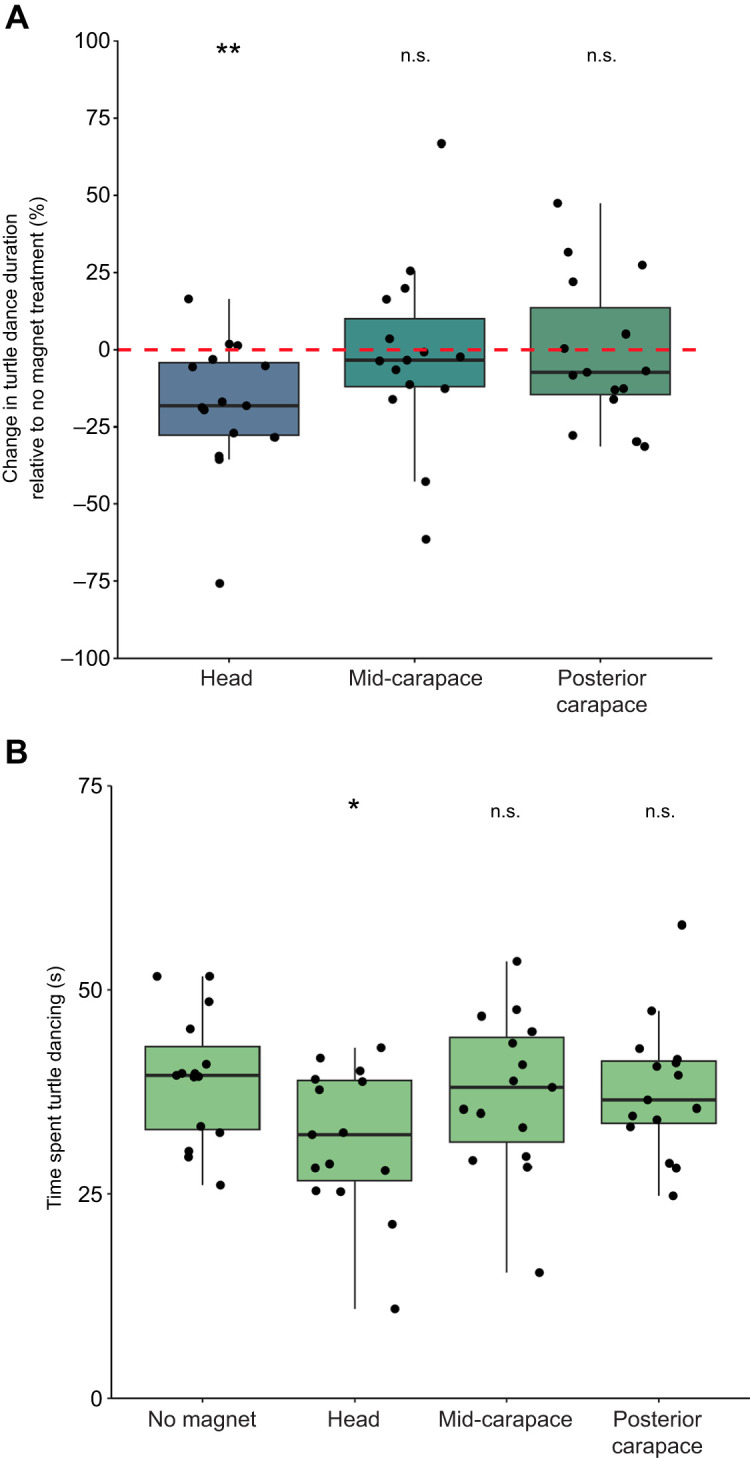
**Effect of magnet treatment on time turtles spent dancing in rewarded field.** (A) Percentage change in turtle dancing time for each magnet treatment relative to the no-magnet control treatment. Turtles with a magnet on their head showed a percentage change significantly less than zero (one-tailed Wilcoxon signed-rank test, *w*=9, *P*=0.001, *n*=15). By contrast, the percentage changes in the mid-carapace and posterior carapace treatments were not significantly different from zero (one-tailed Wilcoxon signed-rank tests, mid-carapace: *w*=52, *P*=0.339, *n*=15; posterior carapace: *w*=51, *P*=0.319, *n*=15). Dashed red line represents a 0% change. The *x*-axis indicates the treatment being compared with the no-magnet control treatment. (B) Mean duration of turtle dancing under control and magnet treatments. Turtles in the head treatment showed a significant reduction in dancing duration (linear mixed-effects model, *t*=−2.64, *P*=0.011, *n*=15), whereas the other treatment groups showed no changes in dancing relative to the control (linear mixed-effects model, mid-carapace: *t*=−0.64, *P*=0.527, *n*=15; posterior carapace: *t*=−0.48, *P*=0.633, *n*=15). The *x*-axis indicates the magnetic treatment. In both A and B, each dot represents results from a single individual. Boxes span the first and third quartiles. Center line indicates the median. Whiskers extend to the most extreme (smallest and largest) points within 1.5-times the interquartile range. n.s., not significant, (*P*>0.05); *P<0.05; **P<0.01.

We then tested the ability of the turtles to recognize the rewarded field with magnets in three different locations. We first calculated the percentage change in dancing duration for each turtle in the three magnet treatments, relative to the duration of dancing in the no-magnet control treatment (Fig. 3A). We expected a zero percentage change if a magnet treatment had no effect, and a negative change (i.e. less than zero) if the treatment did have an effect. The percentage change for turtles with magnets on their heads was significantly lower than zero, indicating a significant decrease in time dancing (Fig. 3A; one-tailed Wilcoxon signed-rank test, *w*=9, *P*=0.001). By contrast, the percentage changes for turtles with magnets on the mid-carapace or on the posterior carapace were not significantly different from zero, indicating that these two treatments did not affect turtle responses (Fig. 3A; one-tailed Wilcoxon signed-rank tests, mid-carapace: *w*=52, *P*=0.339; posterior carapace: *w*=51, *P*=0.319).

Results from the linear mixed-effects model, which relied on duration of turtle dancing rather than percentage increase, reinforced this finding, showing that turtles with the magnet placed on the head displayed a significant reduction in dancing duration (*t*=−2.64, *P*=0.011; Table S2), whereas the other treatments showed no differences in dancing duration relative to the control (mid-carapace: *t*=−0.64, *P*=0.527; posterior carapace: *t*=−0.48, *P*=0.633; Table S2). Thus, in both the linear mixed-effects model and percentage change analysis, a weak magnet placed on the head produced a significant effect, but the same magnet placed on the middle or posterior body did not.

We next conducted pairwise comparisons of the magnet treatments and the control using Tukey's adjustment for multiple comparisons. In Table S3, we report both unadjusted and adjusted *P*-values, which yielded slightly different results. Pairwise comparisons without *P*-value adjustment showed significant differences between no-magnet control and head treatments (*t*=2.642, *P*=0.012) and between head and posterior carapace treatments (*t*=−2.161, *P*=0.036), as well as a marginally significant difference between head and mid-carapace treatments (*t*=−2.004, *P*=0.052), all in keeping with the hypothesis that magnetoreceptors are in the head. By contrast, when *P*-values were adjusted using Tukey's method, the difference between no-magnet control and head treatments became marginally significant (*P*=0.054), and differences between the head treatment and the carapace treatments were non-significant (mid-carapace: *P*=0.203; posterior carapace: *P*=0.151). Whether *P*-values should be adjusted in situations with few, planned comparisons has been debated (Rothman, 1990; Perneger, 1998; Hooper, 2025).

The lack of significant differences when the Tukey correction is applied might reflect a real biological effect if magnetoreceptors are dispersed throughout the body, with most in the head but at least some present near the carapace. If so, then magnets on the carapace might have slightly impaired the magnetic sensitivity of the turtle, just enough that pairwise comparisons could not show a difference between the smaller effects of magnets on the carapace and the larger effects when a magnet was placed on the head.

A statistical limitation, however, also exists. Given that the control does not differ from the carapace treatments, it seems logical that the head treatment, which does differ from the control, should also differ from the carapace treatments – and yet head and carapace treatments are not different. This kind of contradictory pattern is a known consequence of multiple comparison adjustments when statistical power is low (Lee and Lee, 2018). Given our study's low power (0.34) and small sample size (*n*=15), the likelihood that true differences go undetected when *P*-values are adjusted may be unusually high. Thus, the non-significant result when *P*-value adjustments were made is not necessarily evidence of equivalence between effects of the head magnet and the effects of carapace magnets. We therefore conclude that weak magnets placed on the head impair the rewarded response relative to the control and recommend additional work to investigate specifically whether placing a magnet on the head has a greater effect than placing it elsewhere on the body.

Our findings suggest that, in sea turtles, the receptors for the magnetic map sense are located mostly or entirely in the head. An advantage of our protocol is that we used weak magnets which distorted the magnetic field in only a small area; presumably such distortions could only affect receptors near the attachment site. By contrast, previous studies have used much stronger magnets which presumably would have affected large areas of the body (e.g. Keeton, 1971; Sinsch, 1987; Irwin and Lohmann, 2003; Luschi et al., 2007).

An alternative possibility is that the applied magnetic field disrupted neural processing in the brain. This appears unlikely, however, as the weak magnet only altered the ambient magnetic field by several microtesla (Fig. 1B), less than the natural variation a turtle experiences during normal migrations (Lohmann et al., 2012). Moreover, neurons involved in processing signals from primary magnetoreceptors should not differ from other neurons in their sensitivity to magnetic fields. To our knowledge, magnetic fields as weak as those used here have not been reported to impair neural function. We therefore consider the most parsimonious explanation to be that receptors for the magnetic map sense are in the head.

In sea turtles, as well as in birds and salamanders, two different biophysical mechanisms may underlie the magnetic compass and magnetic map (Munro et al., 1997; Wiltschko and Wiltschko, 2007; Phillips and Diego-Rasilla, 2022; Goforth et al., 2025; Mackiewicz et al., 2025). Such a division might evolve if different receptors have become specialized to sense different elements of the magnetic field for different tasks. Thus, receptors for the magnetic compass could be located in a different anatomical area than those for the map. We note, however, that in principle, it is also possible that map and compass information are extracted from the same receptors in some animals (Havens et al., 2025).

The mechanism underlying magnetoreception, either for the compass or the map sense, has not been unequivocally established in any animal. The three hypotheses that have been considered most thoroughly include transduction processes mediated by: (1) the mineral magnetite (Kirschvink et al., 2001); (2) chemical reactions involving radicals and initiated by photoexcitation (Ritz et al., 2000; Hore and Mouritsen, 2016); and (3) electromagnetic induction (Nimpf et al., 2019; Nordmann et al., 2026). In turtles, evidence suggests that the compass sense depends on chemical magnetoreception (Goforth et al., 2025), whereas the map sense uses a different mechanism, possibly magnetite (Mackiewicz et al., 2025). All proposed mechanisms are compatible with cephalic receptors.

Although our results were not aimed at elucidating mechanism, in principle, a magnet should not disrupt magnetoreception based on electromagnetic induction if the magnet does not move relative to the putative sensor. Thus, at first glance, our findings with turtles are incompatible with electromagnetic induction given that the magnet was fastened in place with Velcro and appeared immobile. However, the possibility of slight movements from the fixed position, on the order of 1–2 mm, cannot be excluded. Such movements, if they did occur, raise the possibility of an induced electromotive force that conceivably might disrupt magnetoreception based on electromagnetic induction. For this reason, additional experiments are needed to investigate the induction hypothesis.

Because of the nature of magnetic fields, identifying the physical location of magnetoreceptors has proven difficult. Our results provide evidence that sea turtle magnetic map receptors are in the head, a finding long suspected but never demonstrated. In addition, the method we introduce, of using a weak magnet to generate a magnetic field over a focused area of the body, provides a simple but powerful tool that can be applied to numerous animals. Finally, by using still smaller and weaker magnets, it may be possible in the future to disrupt the field in ever more localized areas of the head (e.g. the eyes versus the ears versus the nose), at least in sea turtles and other animals of sufficient size. This, in turn, may provide further insights into the location and mechanisms of magnetoreceptors.

## Supplementary Material

10.1242/jexbio.252113_sup1 Supplementary information
