# Supplementary material for: Evidence for cephalic magnetic map receptors in sea turtles
Source: J Exp Biol. 2026 May 28;229(10):jeb252113. doi: 10.1242/jeb.252113 (PMC13286376; doi:10.1242/jeb.252113)
Supplement: Supplementary information [file jexbio-229-252113-s1.pdf]

Table S1. Magnetic fields in which turtles were conditioned and tested. Values indicate the average of four measurements made with a Meda tri-axial magnetometer (model FVM-400) in the central area within the coil where turtles were positioned during conditioning and experimental trials. Magnetic field estimates for inclination and intensity for the locations near Virginia, U.S.A. and Newfoundland, Canada were obtained from the International Geomagnetic Reference Field (IGRF) model 13 in spring 2020 using the listed latitude and longitude. The acclimation field approximated the magnetic field that exists in the facility in which turtles were housed, which differed slightly from the natural ambient magnetic field. Declination (the difference between magnetic north and geographic north) was held constant in all magnetic fields because turtles in these experiments do not have access to the necessary celestial cues for determining declination.

Field	Inclination (°)	Intensity (μT)	Latitude/Longitude for field estimate
Virginia, U.S.A.	63.7	50.2	37.4 N, -75.5 W
Newfoundland, Canada	69.6	53.4	49.0 N, -61.8 W
Acclimation	60.4	47.6	Not applicable

Table S2. Linear mixed-effects model results predicting duration of turtle dancing (Avg_Duration) from magnet treatment. Model parameters were estimated using restricted maximum likelihood (REML). Turtle ID was included as a random effect. Conditional $R^2 = 0.25$, Marginal $R^2 = 0.10$. Asterisks represent statistical significance (*P<0.05).

Equation	Fixed Effect (Predictor)	Estimate (β)	95% CI	t-value	p-value
Avg_Duration ~ Treatment + (1|ID)	Intercept (Control)	39.16	[34.68, 43.65]	-	-
	Head	-7.65	[-13.46, -1.84]	-2.64	*0.011
	Mid-Carapace	-1.85	[-7.65, 3.96]	-0.64	0.527
	Posterior Carapace	-1.39	[-7.20, 4.41]	-0.48	0.633

Journal of Experimental Biology • Supplementary information

Table S3. Pairwise differences in mean duration spent turtle dancing across magnet treatments, estimated from a linear mixed-effects model with individual turtle ID as a random effect. Estimated difference represents the difference in mean duration turtle dancing between groups. Both unadjusted and Tukey's adjusted p-values are reported. Asterisks represent statistical significance (*P<0.05); underlined values represent marginal significance (0.05<P<0.10).

Comparison	Estimated difference (s)	t-value	p-value (unadjusted)	p-value (Tukey's adjusted)
Control – Head	7.650	2.642	*0.012	0.054
Control – Mid-Carapace	1.846	0.637	0.527	0.919
Control – Posterior Carapace	1.392	0.481	0.633	0.963
Head – Mid-Carapace	-5.804	-2.004	0.052	0.203
Head – Posterior Carapace	-6.259	-2.161	*0.036	0.151
Mid-Carapace – Posterior Carapace	-0.454	-0.157	0.88	0.999

Journal of Experimental Biology • Supplementary information

Table S4. Duration of time that each individual spent turtle dancing in the rewarded field under each of the four magnet treatments. Reported durations represent an average based on two or more independent observations of the time spent turtle dancing in that treatment.

Turtle ID	Magnet Treatment	Average Duration of Turtle Dancing (s)
L185	No Magnet (Control)	39.77
L185	Head	32.53
L185	Mid-Carapace	15.36
L185	Posterior Carapace	28.76
L186	No Magnet (Control)	39.37
L186	Head	25.38
L186	Mid-Carapace	38.05
L186	Posterior Carapace	36.50
L187	No Magnet (Control)	29.52
L187	Head	27.88
L187	Mid-Carapace	35.38
L187	Posterior Carapace	24.76
L188	No Magnet (Control)	30.24
L188	Head	28.67
L188	Mid-Carapace	28.29
L188	Posterior Carapace	28.17
L189	No Magnet (Control)	51.66
L189	Head	37.75
L189	Mid-Carapace	53.52
L189	Posterior Carapace	35.49
L190	No Magnet (Control)	39.75
L190	Head	32.28
L190	Mid-Carapace	38.84
L190	Posterior Carapace	34.59
L191	No Magnet (Control)	40.90
L191	Head	41.66
L191	Mid-Carapace	47.56
L191	Posterior Carapace	41.06
L192	No Magnet (Control)	48.54
L192	Head	39.04
L192	Mid-Carapace	46.79
L192	Posterior Carapace	34.12
L193	No Magnet (Control)	45.25
L193	Head	10.95
L193	Mid-Carapace	44.92
L193	Posterior Carapace	39.55
L194	No Magnet (Control)	39.32
L194	Head	28.18

Journal of Experimental Biology • Supplementary information

L194	Mid-Carapace	34.88
L194	Posterior Carapace	57.97
L195	No Magnet (Control)	33.30
L195	Head	38.76
L195	Mid-Carapace	29.10
L195	Posterior Carapace	40.63
L196	No Magnet (Control)	32.53
L196	Head	21.32
L196	Mid-Carapace	40.84
L196	Posterior Carapace	42.82
L197	No Magnet (Control)	39.52
L197	Head	40.07
L197	Mid-Carapace	33.15
L197	Posterior Carapace	41.51
L198	No Magnet (Control)	51.67
L198	Head	42.94
L198	Mid-Carapace	29.58
L198	Posterior Carapace	47.41
L200	No Magnet (Control)	26.08
L200	Head	25.27
L200	Mid-Carapace	43.50
L200	Posterior Carapace	33.24

Journal of Experimental Biology • Supplementary information
